# Retraction: Identifying developmental trajectories of worldwide road traffic accident death rates using a latent growth mixture modeling approach

**DOI:** 10.1371/journal.pone.0253092

**Published:** 2021-06-04

**Authors:** The PLOS ONE Editors

After this article [1] was published, concerns were raised that this work was published previously in the Razi Journal of Medical Sciences [2].

The dataset, LGM and LGMM methods, and the following results in [1] appear to duplicate material published previously in [2]:

- Figs 1, 2a, and 2b [1] appear to duplicate results reported in Figs 3 and 4 of [2].
- Fig 3 [1] includes estimated mean growth trajectories previously reported in Fig 5 of [2]. Comparing the two figures it appears that the estimated versus observed trajectories for Class 3 may be mislabelled in the *PLOS ONE* figure.
- The data reported for “Unconditional linear LGM” and “Unconditional Nonlinear LGM” in Table 2 [1] were reported in Table 1 of [2].
- Tables 3 and 4 [1] duplicate results reported in Tables 2 and 3 of [2].
- S1 Fig panel A [1] duplicates Fig 6 in [2].

The 2017 article [2] was published under a CC-BY-NC license, but the *PLOS ONE* article did not discuss, cite, or provide attribution to [2], and did not mention that the above results had been published previously. As such, the republication of the above material in [1] did not comply with the terms of the CC-BY-NC license. The authors apologized for not citing [2] in the *PLOS ONE* article.

The *PLOS ONE* article reported some results that were not reported in [2]. Even so, the *PLOS ONE* Editors concluded that the degree of overlap exceeds what is acceptable and presents concerns regarding redundancy in the literature and adherence to PLOS ONE’s policy on Submission and Publication of Related Studies.

In light of the above concerns, the *PLOS ONE* Editors retract this article.

MS and TM did not agree with retraction. MM and AD either could not be reached or did not respond directly.
